# Rational design of novel coumarins: A potential trend for antioxidants in cosmetics

**DOI:** 10.17179/excli2019-1903

**Published:** 2020-02-26

**Authors:** Apilak Worachartcheewan, Veda Prachayasittikul, Supaluk Prachayasittikul, Visanu Tantivit, Chareef Yeeyahya, Virapong Prachayasittikul

**Affiliations:** 1 Department of Community Medical Technology, Faculty of Medical Technology, Mahidol University, Bangkok 10700, Thailand; 2 Center of Data Mining and Biomedical Informatics, Faculty of Medical Technology, Mahidol University, Bangkok 10700, Thailand; 3 Department of Clinical Microbiology and Applied Technology, Faculty of Medical Technology, Mahidol University, Bangkok 10700, Thailand

**Keywords:** coumarin, antioxidant activity, rational design, QSAR, MLR

## Abstract

Coumarins are well-known for their antioxidant effect and aromatic property, thus, they are one of ingredients commonly added in cosmetics and personal care products. Quantitative structure-activity relationships (QSAR) modeling is an *in silico* method widely used to facilitate rational design and structural optimization of novel drugs. Herein, QSAR modeling was used to elucidate key properties governing antioxidant activity of a series of the reported coumarin-based antioxidant agents (**1**-**28**). Several types of descriptors (calculated from 4 softwares i.e., Gaussian 09, Dragon, PaDEL and Mold^2 ^softwares) were used to generate three multiple linear regression (MLR) models with preferable predictive performance (*Q^2^_LOO-CV_* = 0.813-0.908; *RMSE_LOO-CV_* = 0.150-0.210;* Q^2^_Ext_* = 0.875-0.952; *RMSE_Ext_* = 0.104-0.166). QSAR analysis indicated that number of secondary amines (nArNHR), polarizability (G2p), electronegativity (D467, D580, SpMin2_Bhe, and MATS8e), van der Waals volume (D491 and D461), and H-bond potential (SHBint4) are important properties governing antioxidant activity. The constructed models were also applied to guide *in silico* rational design of an additional set of 69 structurally modified coumarins with improved antioxidant activity. Finally, a set of 9 promising newly design compounds were highlighted for further development. Structure-activity analysis also revealed key features required for potent activity which would be useful for guiding the future rational design. In overview, our findings demonstrated that QSAR modeling could possibly be a facilitating tool to enhance successful development of bioactive compounds for health and cosmetic applications.

## Introduction

Free radicals (or oxidants) are highly reactive molecules containing an unpaired electron, which are generated as by-products of physiological processes and intracellular pathways (Valko et al., 2007[50]; Winyard et al., 2005[52]). These oxidants are well-known for their harmful potential and deleterious effects in cellular components (i.e., DNA, proteins and lipids). In normal condition, these radicals are scavenged/neutralized by antioxidant defense mechanism (i.e., endogenous antioxidant molecules and antioxidant enzymes) to prevent cellular oxidative damages. However, the shift of oxidative balance occurs in a condition whereby radicals are overproduced or antioxidant defense mechanism is depleted. This situation leads to excessive accumulation of free radicals and oxidative stress. Oxidative damage involves in pathogenesis and progression of many chronic and aging diseases (i.e., cancer, diabetes mellitus, neurodegenerative diseases, and cardiovascular diseases) (Valko et al., 2007[50]; Winyard et al., 2005[52]). Furthermore, free radicals have been recognized as one of the factors contributing to aging skin (Bogdan Allemann and Baumann, 2008[5]). Antioxidant compounds have been well-recognized for their wide-ranging health applications, especially in cosmeceutical area. Currently, an addition of antioxidants as active ingredients in cosmetics and personal care products has been widely documented (Kusumawati and Indrayanto, 2013[21]; Lupo, 2001[23]). Therefore, discovery of novel potent antioxidant compounds, both from chemical synthesis (Prachayasittikul et al., 2009[32]; Subramanyam et al., 2017[47]; Worachartcheewan et al., 2012[58]) and natural-derived sources (Elansary et al., 2018[9]; Krishnaiah et al., 2011[20]; Prachayasittikul et al., 2008[30], 2009[31], 2013[36]; Wongsawatkul et al., 2008[55]), has been noted to be an attractive research area, especially in cosmetic applications (Kusumawati and Indrayanto, 2013[21]; Lupo, 2001[23]). 

Coumarins, known as benzopyrones, are natural secondary metabolites bearing fused benzene and *α*-pyrone rings (Witaicenis et al., 2014[53]). Natural-derived coumarins are found in a wide range of plants (Lee et al., 2007[22]; Rodríguez-Hernández et al., 2019[40]; Saleem et al., 2019[44]; Venditti et al., 2019[51]). Coumarins displayed a variety of biological activities including antimicrobial (Arshad et al., 2011[3]), antioxidant (Erzincan et al., 2015[10]), anticancer (Nasr et al., 2014[28]), and anti-inflammatory (Witaicenis et al., 2014[53]) activities. Although synthetic coumarins were banned for oral products due to their potential toxicities, they are attractive for topical uses due to their high skin penetrating property (Stiefel et al., 2017[46]). Additionally, coumarins are widely used as fragrance ingredient in cosmetics and personal care products because of their sweet herbaceous scent (Ma et al., 2015[24]; Stiefel et al., 2017[46]). Antioxidant property and protective effects against skin photo-aging of coumarins have also been remarked in cosmetic area (Kostova et al, 2011[19]; Lee et al., 2007[22]). Previously, a set of synthesized coumarin derivatives containing 2-methylbenzothiazolines, sulphonamides, and amides were reported to exhibit antioxidant activity with IC_50_ values range of 0.024-2.888 mM (Khoobi et al., 2011[18]; Saeedi et al., 2014[42]). However, deeper understanding of structure-activity relationships (SAR) and mechanism of action is still necessary for an effective rational design of coumarin-based antioxidant agents (Kostova et al., 2011[19]). 

Computational approaches have been widely recognized to facilitate and increase success rate of drug development (Nantasenamat and Prachayasittikul, 2015[27]; Prachayasittikul et al., 2015[37]). Quantitative structure-activity relationship (QSAR) modeling is an *in silico* method to reveal the relationship between chemical structures of the compounds and their biological activities. QSAR modeling provides useful findings such as key features, properties, or moieties that are required for potent activity, which would benefit further rational design of the related compounds. Currently, success stories of QSAR-driven rational design of several classes of promising lead compounds have been documented for anticancer agents (Prachayasittikul et al., 2015[33]), aromatase inhibitors (Prachayasittikul et al., 2017[35]), and sirtuin-1 activators (Pratiwi et al., 2019[38]). In cosmetic area, QSAR modeling has been employed to improve understanding towards SAR of tyrosinase inhibitors (Gao, 2018[13]; Khan, 2012[17]). 

**Figure 1:**
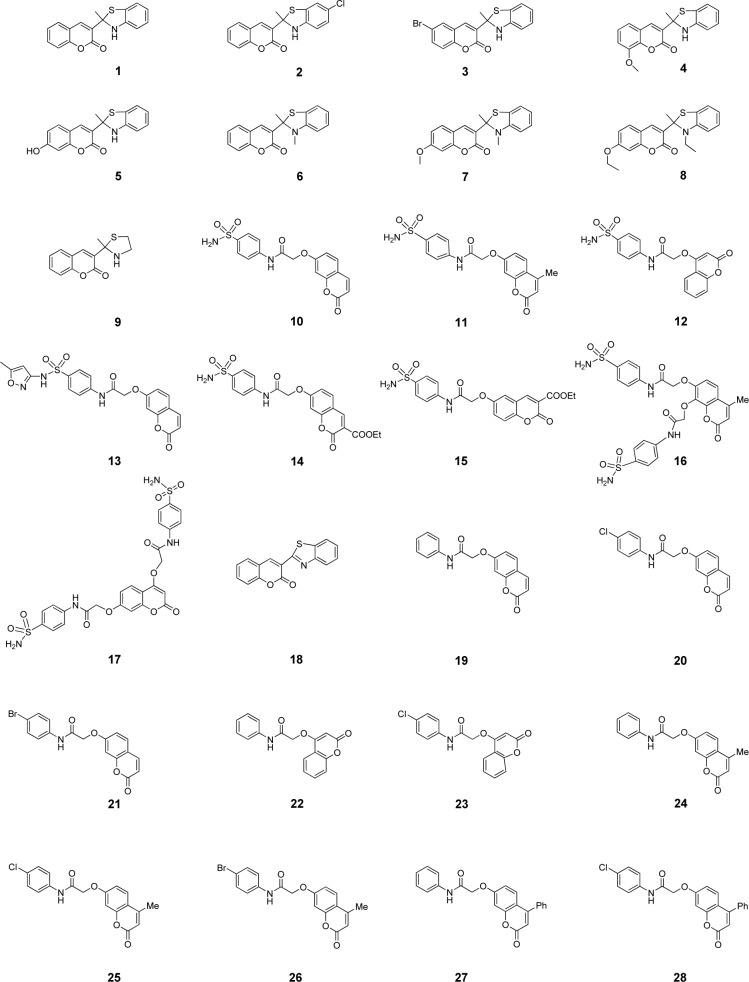
Molecular structures of coumarin derivatives (1-28)

Accordingly, this study aims to construct QSAR models to elucidate SAR of a set of antioxidant coumarin derivatives (**1**-**28**, Figure 1(Fig. 1)) originally reported by Khoobi et al. (2011[18]) and Saeedi et al. (2014[42]). Herein, QSAR models were constructed using multiple linear regression (MLR) algorithm to clearly demonstrate the linear relationship along with insight SAR analysis. In an attempt to find a robust and validating QSAR models, chemical descriptors were generated using different four softwares (i.e., Gaussian 09, Dragon, PaDEL and Mold^2^ softwares) to increase a variety of represented physicochemical properties. Consequently, an additional set of structurally modified compounds were rationally designed based on key findings of the constructed models, and their antioxidant activities were predicted to reveal the promising ones with potential for further synthesis and development. 

## Materials and Methods

### Data set 

**Figure 2:**
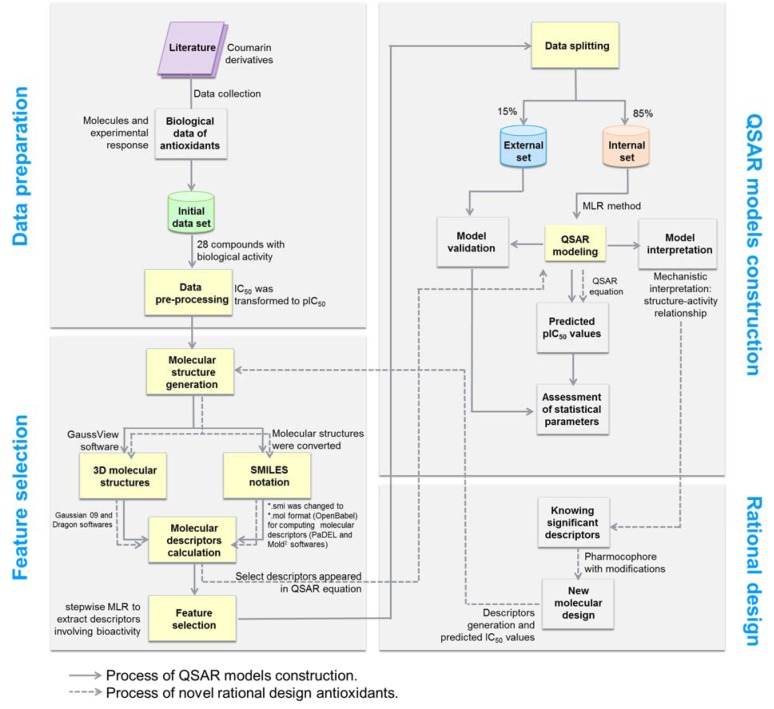
Schematic workflow of QSAR models

A data set of twenty-eight coumarin-based antioxidants (**1**-**28**, Figure 1(Fig. 1)) was retrieved from the literature (Khoobi et al., 2011[18]; Saeedi et al., 2014[42]), in which their antioxidant activities are presented in Table 1(Tab. 1). All tested compounds were evaluated by 1,1-diphenyl-2-picryhydrazyl (DPPH) assay (detailed methodology is provided in original literatures (Khoobi et al., 2011[18]; Saeedi et al., 2014[42])). The activity was denoted as an IC_50_ value (mM) which indicates concentration of the compound which can inhibit 50 % of the generated DPPH radicals in experimental setting. As a part of data pre-processing, the unit of IC_50_ values was converted from mM to M, and the IC_50_ values were further transformed into pIC_50_ (−log IC_50_) by taking the negative logarithm to base 10 as shown in Table 1(Tab. 1). The compound with high pIC_50 _(low IC_50_) represented the high antioxidant activity. A schematic workflow of QSAR model development is provided in Figure 2(Fig. 2).

### Molecular structure optimization

Molecular structures of the coumarin derivatives were constructed by GaussView (Dennington et al., 2003[7]), which were subjected to geometrical optimization by Gaussian 09 (Revision A.02) (Frisch et al., 2009[12]) at the semi-empirical level using Austin Model 1 (AM1) followed by density functional theory (DFT) calculation using Becke's three-parameter hybrid method and the Lee-Yang-Parr correlation functional (B3LYP) together with the 6-31 g(d) basis.

### Descriptor calculation and feature selection

The physicochemical properties (i.e., quantum chemical and molecular descriptors) were generated by different calculating softwares including Gaussian 09, Dragon, version 5.5. (Talete, 2007[49]), PaDEL, version 2.20 (Yap, 2011[60]) and Mold^2^, version 2.0 (Hong et al., 2008[15]) softwares. The calculated descriptors as numerical values could be used to represent properties of the compounds, and were further used as predictors (X variables) for QSAR model construction. List of calculated descriptors are shown as follows.

Quantum chemical descriptors calculation obtained by low energy conformers from the geometrical optimization using Gaussian 09 composed of the total energy (*E_total_*) of the molecule, the highest occupied molecular orbital energy (*E_HOMO_*), the lowest unoccupied molecular orbital energy (*E_LUMO_*), the total dipole moment (μ) of the molecule, the electron affinity (EA), the ionization potential (IP), the energy difference of HOMO and LUMO (HOMO-LUMO_Gap_), Mulliken electronegativity (χ), hardness (η), softness (*S*), electrophilicity (ω), electrophilic index (*ω*_i_), and the mean absolute atomic charge (*Q_m_*) (Worachartcheewan et al., 2014[57]). Furthermore, the output files from geometrical optimization of Gaussian 09 were used as the input data for calculating a set of 3,224 molecular descriptors using Dragon software. The calculated descriptors included 22 classes comprising 48 constitutional descriptors, 119 topological descriptors, 47 walk and path counts, 33 connectivity indices, 47 information indices, 96 2D autocorrelation, 107 edge adjacency indices, 64 burden eigenvalues, 21 topological charge indices, 44 eigenvalue based indices, 41 randic molecular profiles, 74 geometrical descriptors, 150 RDF descriptors, 160 3D-MoRSE descriptors, 99 WHIM descriptors, 197 GETAWAY descriptors, 154 functional group counts, 120 atom centered fragments, 14 charge descriptors, 29 molecular properties, 780 2D binary fingerprints, and 780 2D frequency fingerprints.

An additional set of molecular descriptors was calculated by PaDEL software to give 1,444 1D and 2D descriptors, and Mold^2^ software to generate 777 descriptors by encoding the 2D chemical structure information. Before the calculation, the molecular structures were saved to *.smi and then converted to *.mol files using OpenBabel version 2.3.2 (The Open Babel Package 2015). The *.mol files were used as the input data for calculation by PaDEL and Mold^2^ softwares. 

Descriptors selection was performed to filter a set of important informative descriptors from a whole set of descriptors. Feature selection was initially performed by stepwise multiple linear regression (MLR) using SPSS statistics 18.0 (SPSS Inc., USA) followed by determination of intercorrelation using Pearson's correlation coefficient using cutoff value of |*r*| ≥ 0.9. Any pairs of descriptors with |*r*| ≥ 0.9 were defined as highly correlated predictors, and one of them was excluded.

### Data splitting 

The data set of coumarin derivatives (**1**-**28**) was randomly selected, in which 85 % (~23 compounds) of the original data set was used as the training and the leave one-out cross-validation (LOO-CV) sets, and 15 % (~5 compounds) was used as the external set. The training set was employed to generate the QSAR models, whereas LOO-CV and external sets were used to evaluate the models. LOO-CV method was performed for internal validation by excluding one sample out from the whole data set to be used as the testing set while the remaining *N−1* samples were used as the training set (Prachayasittikul et al., 2014[34]). This sampling process was repeated iteratively until every sample in the data set was used as the testing set. The external sets were used to validate the models. 

### Multivariate analysis

QSAR models were generated using the MLR according to the equation 1.


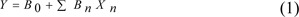


where *Y* is the antioxidant activity (pIC_50_), *B*_0 _is the intercept, and *B_n_* are the regression coefficients of the descriptors *X_n_*. The MLR method was performed using Waikato Environment for Knowledge Analysis (Weka), version 3.4.5 (Witten et al., 2011[54]). 

### Models evaluation

Statistical parameters were used to evaluate predictive performance of the constructed QSAR models. The calculated parameters included correlation coefficient (*R^2^*), root mean squared error (*RMSE*), predictivity (*Q^2^*), variance ratio (*F ratio*), adjusted correlation coefficient (*R^2^_Adj_*), standard deviation (*s*) and predicted residual sum of squares (PRESS) (Saghaie et al., 2013[43]; Worachartcheewan et al., 2013[56]).

## Results and Discussion

### Molecular descriptors selection

Chemical structures of the compounds and their antioxidant activities (Table 1(Tab. 1)) were used for construction of predictive models. The compounds were geometrically optimized with semi-empirical method AM1 followed by DFT/B3LYP/6-31 g(d) basis using Gaussian 09 to obtain lower-energy conformers. The optimized compounds were extracted to obtain 13 quantum chemical descriptors. These compounds were subsequently used as input files for calculating an additional set of 3,224 molecular descriptors (0D-3D) using Dragon software. The calculated descriptors with constant values and multi-collinearity were determined and removed to give a final set of 1,489 descriptors. In addition, original molecular structures of compounds were saved as *.smi file format and were converted into *.mol files using OpenBabel version 2.3.2. These *.mol files then were used as input files for descriptors calculation using Mold^2 ^and PaDEL softwares to obtain sets of 777 Mold^2^ 2D descriptors and 1,444 PaDEL 0D-2D descriptors, respectively. Consequently, feature selection was performed to select a set of informative descriptors for the whole calculated set. Descriptors showing significant correlation with their bioactivities were selected using stepwise MLR. A set of 14 informative descriptors included 4 Dragon descriptors (i.e., nArNHR, ISH, B04[O-O] and G2p), 6 Mold^2 ^descriptors (i.e., D467, D278, D491, D384, D580 and D461), 4 PaDEL descriptors (i.e., SHBint4, SpMin2_Bhe, MATS8e and SssCH2) were obtained. Definition and numerical values of important descriptors are shown in Tables 2(Tab. 2) and 3(Tab. 3), respectively. Furthermore, the intercorrelation matrix between pair of molecular descriptors was performed using Pearson's correlation coefficient (*r*) (Supplementary Tables 1-3). Cutoff value of |*r*| ≥ 0.9 was used to determine the intercorrelation. The results showed that there was no intercorrelation within a set of selected descriptors as displayed by low |*r*| values ≤ 0.9, which suggested that each descriptor was independent from other descriptors. Finally, a set of 14 selected descriptors was further employed to construct 3 QSAR models (according to types of software used to calculate descriptor values) for predicting antioxidant activity of the coumarin derivatives.

### QSAR models

Descriptors obtained from these softwares have been demonstrated for their successful QSAR modeling such as antioxidant (Alisi et al., 2018[1]; Rastija et al., 2018[39]), antimicrobial (Alyar et al., 2009[2]; Basic et al., 2014[4]; Podunavac-Kuzmanović et al., 2009[29]), anticancer (Sławiński et al., 2017[45]; Suvannang et al., 2018[48]) and antiviral (Duchowicz et al., 2018[8]; Saavedra et al., 2018[41]; Worachartcheewan et al., 2019[59]) activities. Herein, three models were separately constructed based on the types of key descriptors (i.e., model 1 Dragon descriptors, model 2 Mold^2^ descriptors, and model 3 PaDEL descriptors). A set of 14 selected informative descriptors (as independent variables, Table 2(Tab. 2)) and antioxidant activities (pIC_50 _values as dependent variables) of the studied compounds were included in the data sets for construction of QSAR models using Eq. (1). Before building the models, the data set of coumarin derivatives (**1**-**28**) was split into training, LOO-CV, and external sets. The training set was used to construct the model using MLR algorithm whereas both LOO-CV and external sets were utilized for validating the constructed models. Compounds **1**, **6**, **15**, **21 **and **27 **were randomly selected to be used as external sets, while the remaining 23 compounds in the data sets (i.e., **2**-**5**, **7**-**14**, **16**-**20**, **22**-**26** and **28**) were employed as training set. As a result, three QSAR models (models 1-3) were successfully constructed for predicting antioxidant activities (pIC_50 _values) of the studied coumarin analogs. 

**Model 1** (Dragon descriptors)


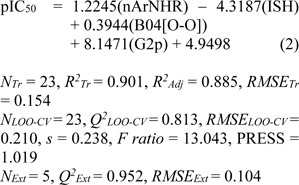


where *N_Tr_*, *N_LOO-CV_* and *N_Ext_* are the number of compounds of training, LOO-CV and external sets. *R^2^_Adj_* is the adjusted *R^2^*.

**Figure 3:**
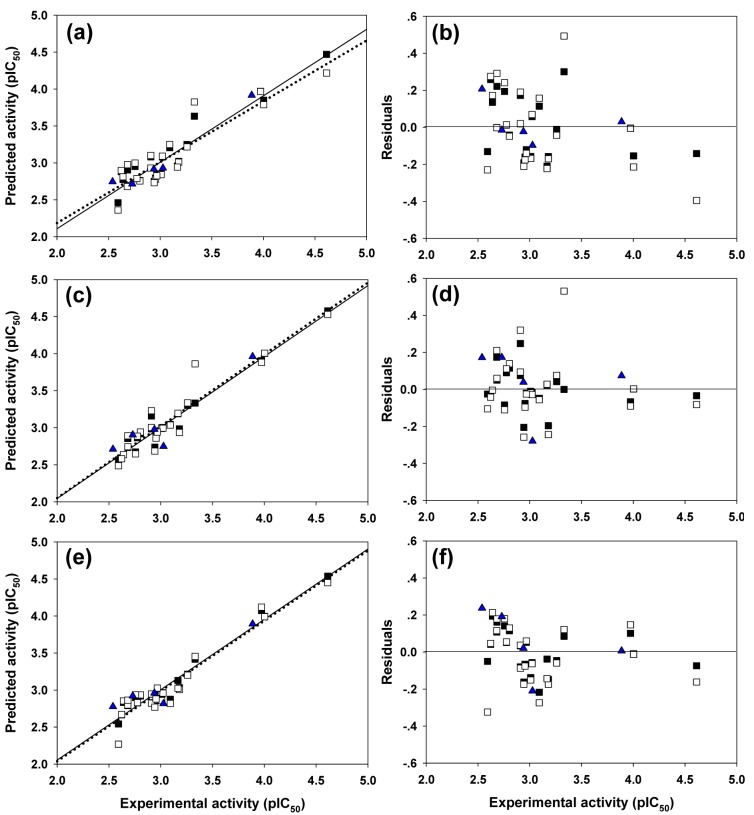
Plots of the experimental and the predicted activities of model 1 (3a), model 2 (3c), and model 3 (3e) for the training set (□; regression line is represented as solid line), the leave-one out cross-validated (■; regression line is represented as dotted line), and the external (▲) sets. Distribution of the experimental activity and residual values (the difference between experimental and predicted activities) of model 1 (3b), model 2 (3d) and model 3 (3f)

Four molecular descriptors calculated from Dragon software were used as predictors to construct QSAR model 1 as shown in Eq. (2). Statistical parameters indicating predictive performance of the model are summarized in Table 4(Tab. 4). Training set showed *R^2^_Tr _*= 0.901, *RMSE_Tr _*= 0.154, while the LOO-CV set displayed the *Q^2^_LOO-CV _*= 0.813, *RMSE_LOO-CV _*= 0.210, and the external set with *Q^2^_Ext_* = 0.952, *RMSE_Ext_* = 0.104. Comparative plot of experimental versus predicted antioxidant activities (pIC_50_) is shown in Figure 3a(Fig. 3). Residual values were calculated as a difference between experimental and predicted pIC_50_ values. Small residual values indicated the precision of model prediction. Plot of the residual values is shown in Figure 3b(Fig. 3).

**Model 2** (Mold^2^ descriptors)


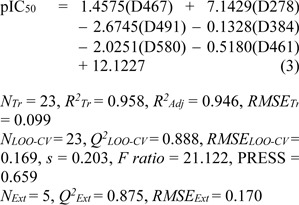


Six important descriptors obtained from the Mold^2^ software were used to generate the model 2 as shown in Eq. (3). Statistical analysis of the model is given in Table 4(Tab. 4). It was observed that the model provided statistical *R^2^_Tr _*= 0.958, *RMSE_Tr _*= 0.099 for the training set, *Q^2^_LOO-CV _*= 0.888, *RMSE_LOO-CV _*= 0.169 for the LOO-CV set, and *Q^2^_Ext_* = 0.875, *RMSE_Ext_* = 0.170 for the external set. The experimental versus the predicted antioxidant activities of the model 2 were graphically plotted (Figure 3c(Fig. 3)), while Figure 3d(Fig. 3) was the plot of residual values. 

**Model 3** (PaDEL descriptors)


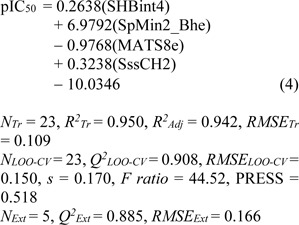


Model 3 of Eq. (4) was built using 4 significant descriptors generated by PaDEL software. Statistical evaluation of the model is shown in Table 4(Tab. 4). The results showed that the QSAR model displayed *R^2^_Tr _*= 0.950, *RMSE_Tr _*= 0.109 for the training set, *Q^2^_LOO-CV _*= 0.908, *RMSE_LOO-CV _*= 0.150 for the LOO-CV set, and *Q^2^_Ext_* = 0.885, *RMSE_Ext_* = 0.166 for the external set. Comparison of experimental and the predicted antioxidant activities of model 3 are outlined in Figure 3e(Fig. 3), while residual values were displayed in Figure 3f(Fig. 3). 

In overview, three constructed models provided satisfactory results as indicated by their statistical parameters such as *R^2^, Q^2^, RMSE, F ratio* and PRESS values. The *R^2^* and* Q^2^* of the obtained QSAR models were considered as acceptable values when *R^2^*>0.6 and *Q^2^*>0.5 (Golbraikh and Tropsha, 2002[14]; Nantasenamat et al., 2010[26]). These parameters of all constructed models were in acceptable range (model 1: *R^2^_Tr _*= 0.901,* Q^2^_LOO-CV _*= 0.813 and *Q^2^_Ext_* = 0.952, model 2: *R^2^_Tr _*= 0.958, *Q^2^_LOO-CV _*= 0.888 and *Q^2^_Ext_* = 0.875, model 3:* R^2^_Tr _*= 0.945, *Q^2^_LOO-CV _*= 0.908 and *Q^2^_Ext_* = 0.885. In addition, low values of RMSE, *s* and PRESS, but high value of *F ratio* indicated that the models were significant (*RMSE_Tr _*= 0.154, *RMSE_LOO-CV _*= 0.210, *RMSE_Ext_* = 0.104, *s* = 0.238, PRESS = 1.019 and *F ratio* = 13.043 in model 1, *RMSE_Tr _*= 0.099, *RMSE_LOO-CV _*= 0.169, *RMSE_Ext_* = 0.170, *s* = 0.203, PRESS = 0.659 and *F ratio* = 21.122 in model 2, and *RMSE_Tr _*= 0.109, *RMSE_LOO-CV _*= 0.151, *RMSE_Ext_* = 0.164, *s* = 0.170, PRESS = 0.518 and *F ratio* = 44.52 and in model 3) (Frimayanti et al., 2011[11]; Rastija et al., 2018[39]). The statistical (Table 4(Tab. 4)) and graphical (Figure 3(Fig. 3)) results showed that the QSAR models (models 1-3) gave a reliable agreement of the experimental and the predicted antioxidant values. Furthermore, the plots of experimental activity and residual values (Figures 3b, 3d and 3f(Fig. 3)) displayed the distribution of residuals on both sides of the zero values indicating that there are no systemic error in the models (Jalali-Heravi and Kyani, 2004[16]). Therefore, the QSAR models 1-3 could be possibly used and reliable for predicting the antioxidant activity of coumarin derivatives. Considering the correlation coefficient (*Q^2^*) of external set, it was shown that the Dragon descriptors gave the highest quality of the prediction for external test set (model 1: *Q^2^_Ext_* = 0.952) followed by the PaDEL descriptors (model 3: *Q^2^_Ext_* = 0.885) and the Mold^2^ descriptors (model 2: *Q^2^_Ext_* = 0.875). 

### Structure-activity relationship (SAR)

Regression coefficient values of the key descriptors (as independent variables) in QSAR models define the degree or weight of their influence on dependent variables (antioxidant activity: pIC_50 _values). According to the linear QSAR equations, high values of descriptors with positive regression coefficient and low values of descriptors with negative regression coefficient are required for potent antioxidant activity (high pIC_50 _values).

The descriptors in model 1 including nArNHR, B04[O-O] and G2p displayed positive values of regression coefficient involved in the increased antioxidant activity as a positive effect, while ISH descriptor having negative value of regression coefficient involved in the decreased activity as a negative effect. The important descriptors are ranked according to their regression coefficient values as G2p>nArNHR>B04[O-O]>ISH with corresponding values of 8.1471, 1.2245, 0.3944, and -4.3187, respectively. The descriptors in model 2 including D467 and D278 with positive regression coefficient values displayed the positive effect, whereas D491, D384, D580 and D461 descriptors exerted the negative effect on the bioactivity. The order of descriptors are D278>D467>D384>D461> D580>D491 with corresponding values of 7.1429, 1.4575, -0.1328, -0.5180, -2.0251, and -2.6745, respectively. For model 3, SHBint4, SpMin2_Bhe and SssCH2 descriptors showed the positive effect on activity, but MATS8e descriptor displayed the negative effect. The order of important descriptors are SpMin2_Bhe>SssCH2> SHBint >MATS8e with values of 6.9792, 0.3238, 0.2638 and -0.9768, respectively. 

To gain insights into SAR, coumarin derivatives (**1**-**28**, Figure 1(Fig. 1)) are categorized into 3 groups according to their core structures (i.e., thiazole group I (**1**-**9** and **18**), sulfonamides group II (**10**-**17**) and amides group III (**19**-**28**) for effective SAR analysis. Thiazoles group I (**1**-**9** and **18**) showed antioxidant activity (Table 1(Tab. 1)) with pIC_50_ range of 2.539-4.612. The most potent and the least potent compounds of benzothiazoles group I were **5** (pIC_50_ = 4.612) and **6 **(pIC_50_ = 2.539), respectively. Among group II compounds (**10**-**17**), compound **11** was the most active (pIC_50_ = 3.180), and **14** was the least active compound. For group III of amides **19**-**28**, compound **21** displayed the most potent activity (pIC_50_ = 3.027) and compound **19** exhibited the lowest activity with pIC_50_ of 2.640. 

It should be noted that coumarins substituted by thiazole at 3-position displayed better activity when compared with those substituted by sulfonamides and amides at 7- or 4- or 6- position (group II and III). The antioxidant activity (Table 1(Tab. 1)) is shown as the following trend: group I **5**>**3**>**4**>**1**>**2**>**18**>**8**>**7**>**9**>**6**; group II **11**>**16**>**13**>**17**>**15**>**12**>**10**>**14**; group III **21**>**28**>**22**>**20**>**23**>**26**>**27**>**24**>**25**>**19**.

According to the significant descriptors in models 1-3, secondary (*sec*-) amine, polarizability, electronegativity and H-bond displayed positive effect in the antioxidant activity. This is noted in the most potent coumarin **5** bearing *sec*-amine (part of aromatic thiazole), and 7-OH group (on the coumarin ring) with H-bond and polarizability properties. On the other hand, tertiary (*tert*-) amine **6** without 7-OH group exerted the lowest activity among the coumarin derivatives **1**-**28**. This could be implied that the *sec*-amine (-NH-) and OH as H-bond and polarizing group are important for the better activity. 

In a series of compounds **1**-**28**, only the *sec*-amines (**1**-**5**) had nArNHR = 1. The most potent compound **5** had higher values of nArNHR = 1, SHBint4 = 7.5875, G2p = 0.193, D467 = 0.805, but lower values of van der Waals volume (D491 = 0.471 and D461 = 0.186) when compared with the least potent compound **6** (nArNHR = 0, SHBint4 = 0.000, G2p = 0.169, D467 = 0.646, van der Waals volume: D491 = 0.805 and D461 = 0.520). Apparently, the *tert*-amines (**6**-**8**) and other amides, sulfonamides (**10**-**28**) as well as non-aromatic *sec*-amine **9** had nArNHR = 0.

**Figure 4:**
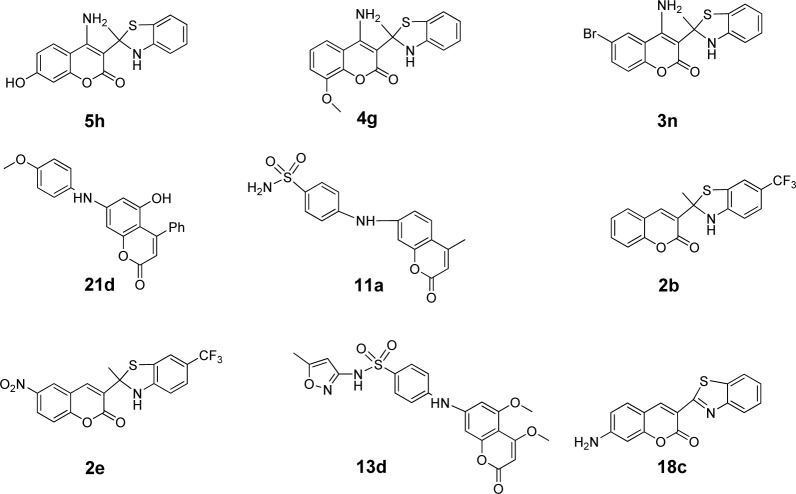
The most potent compounds in each rationally modified series

Because each descriptor represents certain characteristic/property of the compound, QSAR equations are useful to guide effective rational molecular design of novel bioactive compounds with preferable activity (De et al., 2017[6]; Mitra et al., 2011[25]; Prachayasittikul et al., 2017[35]; Pratiwi et al., 2019[38]). To improve the antioxidant activity of these derivatives (**1**-**28**), 8 compounds (i.e., **2**, **3**, **4**, **5**, **11**, **13**, **18** and **21**) were selected as parent compounds for structural modification. A series of novel analogs were rationally designed based on key influencing properties (which were revealed by descriptors presented in models 1-3). The modification was conducted by substitution of diverse types of functional groups with electronegativity, polarizability and H-bond properties (i.e., electron donating and electron withdrawing groups such as OH, NH_2_, SH, OCH_3_, CN, CF_3_ and halogen) on various positions of the coumarin ring. The new structurally modified compounds are shown in Supplementary Table 4). Values of molecular descriptors of these compounds are provided in Supplementary Tables 5-7). The prediction showed that the most potent compounds (pIC_50_) of each modified series were **2b**, **2e **(4.503, 4.502), **3n** (6.340), **4g** (6.445), **5h** (7.016), **11a** (4.526), **13d** (4.356), **18c** (4.197) and **21d** (5.634) as shown in Figure 4(Fig. 4). All highly potent modified compounds were ranked as **5h**>**4g**>**3n**>**21d**>**11a**>**2b= 2e**>**13d**>**18c**. The top three modified compounds (ranked as **5h**>**4g**>**3n**) shared a common feature of 4-amino coumarin moiety (Figure 4(Fig. 4)). This moiety involves in H-bonding, polarizability, and electronegativity properties of the compounds which are essential for potent predicted activity. Apparently, these most potent compounds displayed the highest SHBint4 values of 16.903, 13.660, and 13.064 for **5h**, **4g**, and **3n**, respectively.

The following discussed compounds are shown in Supplementary Table 4. Compound **2** (pIC_50_ = 3.332) was modified by substitution of R1 (6-position) and R2 (5-position) groups (H, F, Cl, CF_3_, CN, NO_2_) on the core structure to give a new series of compounds **2a**-**2h **with improved activity (pIC_50_ = 3.167-4.503), except for compounds **2f **and **2h**. Compounds **2b **(5-CF_3_, 6-H) and **2e** (5-CF_3_, 6-NO_2_) were the most potent compounds with comparable pIC_50 _values of 4.503 and 4.502, respectively. These could be due to the enhancing effect of 5-CF_3 _(R2) and 6-NO_2_ (R1) groups that provided high polarizability (G2p: **2b** = 0.1820, **2e **= 0.1730), electronegativity (SpMin2_Bhe: **2b** = 1.847, **2e **= 1.847), and potential H-bond (SHBint4:** 2b** = 5.150, **2e **=5.480), thus, improved antioxidant activity of the modified compounds comparing with their parent **2** (G2p = 0.164, SpMin2_Bhe = 1.8175, SHBint4 = 4.0816).

Similarly, modified compounds **3a**-**3p** were obtained by substitution of R1, R2, R3 at positions 7, 6, and 4 of the parent compound **3** (R1 = H, OH, OCH_3_, NH_2_, N(CH_3_)_2_, SH, SC_6_H_5_; R2 = F, Cl, Br, CF_3_; R3 = H, OH, OCH_3_, NH_2_, N(CH_3_)_2_). Compound **3n** (R1 = H, R2 = Br, R3 = NH_2_) was predicted as the most potent one with pIC_50_ value of 6.340. Compounds **3f**-**3p** (pIC_50_ = 4.129-6.340) were mostly active than the parent compound** 3** (pIC_50 _= 4.003). It should be noted that these compounds (**3f**-**3p**) contained the same type of substituted R2 (Br) group as their parent **3** (6-Br). Therefore, higher H-bonding potential (SHBint4 = 13.064) and electronegativity (D467 = 0.736) play an important role in improving the activity of compound **3n** when compared with its parent compound (**3**: SHBint4 = 4.2075, D467 = 0.722). The higher SHBint4 and D467 of **3n** may result from the property of the substituted NH_2_ group in forming H-bond with the interacting species. In addition, the substitution with high electronegativity Br group (R2) at position 6 is required for improving activity of the compounds as noted for compound** 3f-3p** and the parent compound **3**.

**Figure 5:**
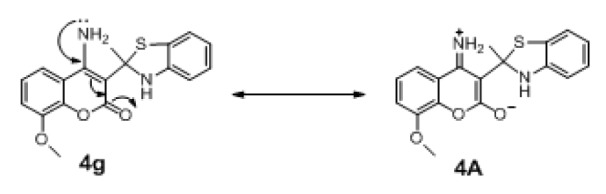
The resonant ionic form of 4A

Structural modification improved activity of most of the compounds in series **4 **(**4a**-**4i**: pIC_50_ = 4.033-6.445), except for compounds **4d** and **4h**.The results showed that **4g** (R1 = 7-H, R2 = 4-NH_2_) was the most potent compound (pIC_50_ = 6.445). Comparing with the parent compound **4** (pIC_50_ = 3.973, SHBint4 = 4.442, G2p = 0.174), the most potent derivative **4g** displayed higher potential H-bond (SHBint4 = 13.660) and higher polarizability (G2p = 1.960). It is reasonable to explain that 4-NH_2_ (R2) of **4g** participates in H-bond forming and polarization through a keto group of the coumarin ring as shown by the resonant ionic form (**4A**) in Figure 5(Fig. 5). On the other hand, compound **4c** (R1 = 7-NH_2_, R2 = 4-H) exerted lower activity with lower descriptors values (pIC_50 _= 0.4772, SHBint4 = 7.432, G2p = 0.1830) comparing with the most potent compound **4g**.

The most potent coumarin **5** was modified to give derivatives **5a**-**5l**, in which compounds **5f**-**5i **displayed the improved antioxidant activity (pIC_50_ = 4.745-7.016). Compound **5h** was the most potent one (pIC_50_ = 7.016) with higher H-bond potential (SHBint4 = 16.903), but lower electronegativity (MATS8e = 0.063) when compared with its parent **5** (pIC_50_ = 4.612, SHBint4 = 7.5875, MATS8e = 0.0848).

The amides **11**, **13**, and **21** were structurally modified by removing oxyketo group from the parent core structures to give *sec-*amines. The amine derivatives **11a**-**11f** with improved activity (pIC_50_ = 3.402-4.526) were achieved from compound **11** (pIC_50_ = 3.180). When compared with its parent (amide **11**: nArNHR = 0, D467 = 0.479, SHBint4 = 0.000, D491 = 0.688, D461 = 0.544 and MATS8e = 0.1872), improved activity of the most potent amine **11a** (pIC_50_ = 4.526) was governed by its higher nArNHR = 1, electronegativity (D467 = 0.690), and H-bond (SHBint4 = 2.048), but lower van der Waals volume (D491 = 0.342 and D461 = 0.022) and electronegativity (MATS8e = -0.058). This could suggest that the improved activity of compounds requires a smaller size *sec-*amine (**11a**) with lower van der Waals volume but higher potential H-bonding. Similarly, a series of *sec-*amines **13a**-**13d **(R1 = CH_3_, OH, OCH_3_, R2 = CH_3_, OCH_3_) were obtained (pIC_50_ = 3.341-4.356) from the amide **13** (pIC_50_ = 3.022). Compound **13d** (pIC_50_ = 4.356) was the most potent amine with higher nArNHR = 1, D467 = 0.640, SHBint4 = 4.368, but lower van der Waals volume D461 = 0.455 and MATS8e = -0.125 comparing to its parent compound **13** (nArNHR = 0, D467 = 0.503, SHBint4 = 0.000, D461 = 0.515 and MATS8e = 0.1400). The amide **21** (pIC_50_ = 3.027) was modified to give analogs with improved antioxidant effect i.e., *sec*-amines analogs **21a**-**21h** (pIC_50_ = 3.549-5.634). The most potent modified amine **21d** (pIC_50_ = 5.634) displayed higher nArNHR = 1, electronegativity D467 = 0.657, H-bond SHBint4 = 9.823, electronegativity SpMn2-Bhe = 1.843 and SssCH_2_ = 0, but lower ISH = 0.869, van der Waals volume D491 = 0.350 and D461 = 0.436, and MATS8e = -0.219 when compared with the parent **21** (nArNHR = 0, D467 = 0.489, SHBint4 = 0.000, SpMin2-Bhe = 1.8395, SssCH_2_ = -0.1139, ISH = 0.923, van der Waals volume D491 = 0.82 and D461 = 0.612, and MATS8e = -0.0511).

Benzothiazole coumarin **18** was structurally modified to provide compounds **18a**-**18f** (R1 = OH, OCH_3_, NH_2_, N(CH_3_)_2_, SH, SC_6_H_5_ at position 7). Comparing with the parent compound **18** (pIC_50_ = 3.260), compounds **18b**-**18e** were more potent antioxidants (pIC_50_ = 3.758-4.197). Improved activity of the most potent compound **18c** (pIC_50_ = 4.197) was governed by higher electronegativity (D467 = 0.595), total information content (D278 = 0.473) and H-bond (SHBint4 = 2.894), but lower van der Waals volume (D491 = 0.377and D461 = 0.299) when compared to parent compound **18** (D467 = 0.501, D278 = 0.413, SHBint4 = 0.000, D491 = 0.472 and D461 = 0.471).

It should be noted that the most potent modified compounds had higher values of H-bonding descriptor (SHBint4 = 2.048-16.903, Supplementary Table 7) when compared with their parent compounds (SHBint4 = 0.000-7.5875, Table 3(Tab. 3)). Thus, SHBint4 might be the important descriptor in governing the potent antioxidant activity.

## Conclusion

Understanding SAR is important for improving bioactivities and pharmacokinetic properties in development of potent and safe cosmetic products. Herein, a set of coumarin derivatives (**1**-**28**) with antioxidant activity was used to construct three QSAR models (1-3) using three different descriptor types and MLR method. Results of statistical evaluation showed that three generated QSAR models provide good reliability and comparable predictive performance (*Q^2^_LOO-CV_* = 0.813-0.908; *RMSE_LOO-CV_* = 0.150-0.210;* Q^2^_Ext_* = 0.875-0.952; *RMSE_ Ext_* = 0.104-0.166). In addition, good correlation obtained from model prediction suggests that the selected significant descriptors were shown to be good representatives for revealing correlation between chemical structures of the compounds (i.e., nArNHR, H-bonding, polarizability, van der Waals volume and electronegativity properties) and their antioxidant activities. An application of the constructed models was demonstrated by rationally designed an additional set of 69 structurally modified coumarins based on key descriptors, in which their antioxidant activities were predicted using the obtained QSAR models (1-3). Most of the rationally designed compounds displayed more improved antioxidant activity when compared with their parents. Particularly, the top three newly designed compounds (**5h**, **4g** and **3n**) showing high H-bonding (SHBint4) descriptor values which may play part in governing the most improved antioxidant activity. Finally, a set of newly designed promising coumarin analogs were highlighted for their potential to be further developed as potent antioxidants. Insights SAR findings also provided beneficial guidelines for the rational design of novel coumarin-based compounds with potent antioxidant effect for cosmetic applications.

## Supplementary information

Supplementary information is available on the EXCLI Journal website.

## Notes

Apilak Worachartcheewan and Supaluk Prachayasittikul (Center of Data Mining and Biomedical Informatics, Faculty of Medical Technology, Mahidol University, Bangkok 10700, Thailand; Phone: (662) 441-4376, Fax: (662) 441-4380, supaluk@g.swu.ac.th) contributed equally as corresponding authors.

## Conflict of interest

The authors declare that they have no conflict of interest.

## Acknowledgements

This work was supported by the Thailand Research Fund (Grant No. MRG6180053) and the annual budget grant from Mahidol University (B.E. 2562-2563).

## Supplementary Material

Supplementary information

## Figures and Tables

**Table 1:**
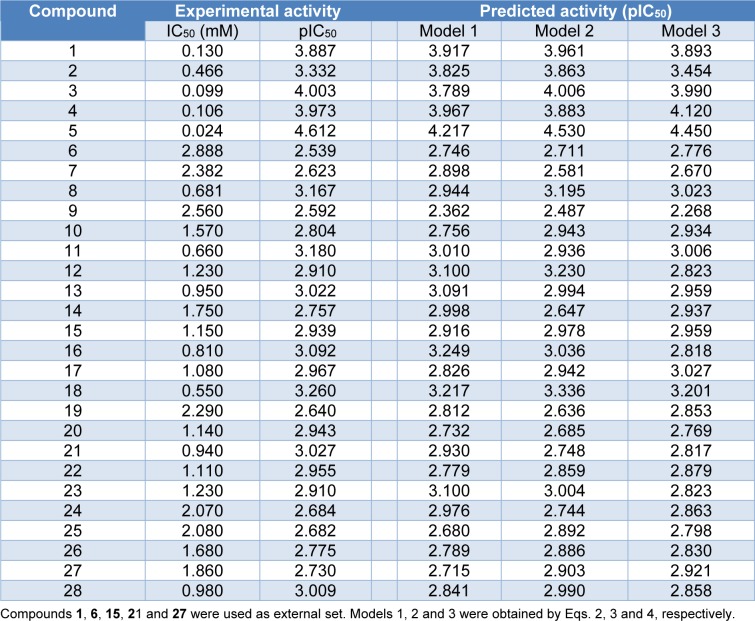
Experimental and predicted antioxidant activities (pIC_50_) of coumarin derivatives (1-28) using multiple linear regression method

**Table 2:**
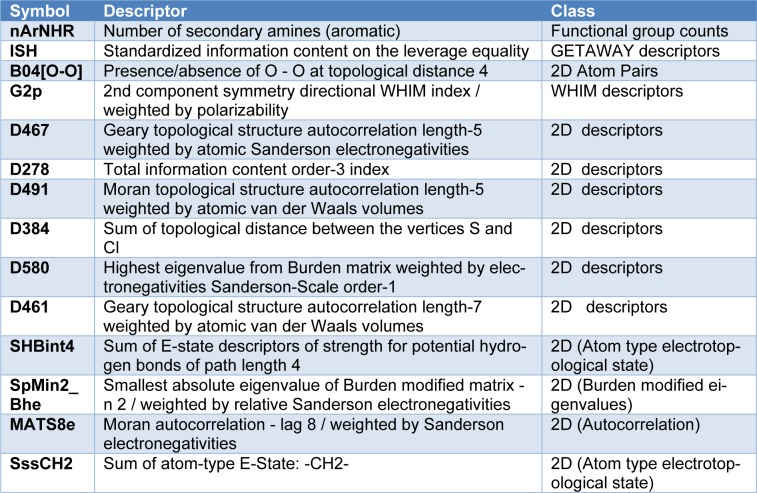
Definition of descriptors in QSAR models (1-3)

**Table 3:**
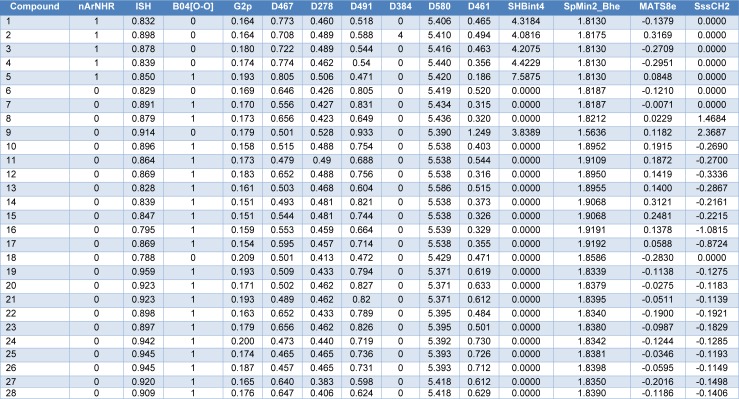
Values of significant molecular descriptors for QSAR models (1-3)

**Table 4:**
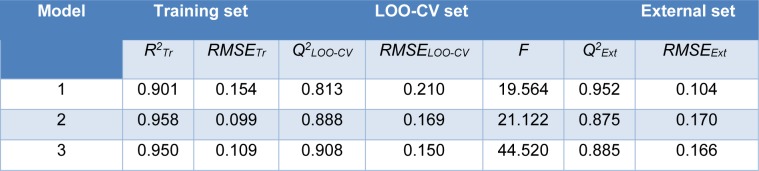
Summary of statistical results in predicting antioxidant activity of coumarin derivatives (1-28)
