# Supplementary material for: Rational design of novel coumarins: A potential trend for antioxidants in cosmetics
Source: EXCLI J. 2020 Feb 26;19:209–26. doi: 10.17179/excli2019-1903 (PMC7105943; doi:10.17179/excli2019-1903)
Supplement: Supplementary information [file EXCLI-19-209-s-001.pdf]

EXCLI Journal 2020;19:209-226 – ISSN 1611-2156
Received: October 18, 2019, accepted: February 19, 2020, published: February 26, 2020

Supplementary information to:

RATIONAL DESIGN OF NOVEL COUMARINS: A POTENTIAL TREND FOR ANTIOXIDANTS IN COSMETICS

Apilak Worachartcheewan[1*], Veda Prachayasittikul[2], Supaluk Prachayasittikul[2*], Visanu Tantivit[1], Chareef Yeeyahya[1], Virapong Prachayasittikul[3]

[1] Department of Community Medical Technology, Faculty of Medical Technology, Mahidol University, Bangkok 10700, Thailand
[2] Center of Data Mining and Biomedical Informatics, Faculty of Medical Technology, Mahidol University, Bangkok 10700, Thailand
[3] Department of Clinical Microbiology and Applied Technology, Faculty of Medical Technology, Mahidol University, Bangkok 10700, Thailand

* Corresponding authors: E-mail: apilak.woa@mahidol.ac.th (A.W), supaluk@g.swu.ac.th (S.P.); Phone: (662) 441-4376; Fax: (662) 441-4380

http://dx.doi.org/10.17179/excli2019-1903

This is an Open Access article distributed under the terms of the Creative Commons Attribution License (http://creativecommons.org/licenses/by/4.0/).

EXCLI Journal 2020;19:209-226 – ISSN 1611-2156
Received: October 18, 2019, accepted: February 19, 2020, published: February 26, 2020

Supplementary Table 1: Intercorrelation matrix of significant molecular descriptors from Dragon software

	nArNHR	ISH	B04[O-O]	G2p
nArNHR	1.000			
ISH	-0.173	1.000		
B04[O-O]	-0.592	0.233	1.000	
G2p	0.089	0.176	-0.229	1.000

Supplementary Table 2: Intercorrelation matrix of significant molecular descriptors from Mold^2 software

	D491	D278	D467	D384	D580	D461
D491	1.000					
D278	0.117	1.000				
D467	-0.502	0.096	1.000			
D384	-0.198	0.189	0.262	1.000		
D580	-0.108	0.223	-0.121	-0.123	1.000	
D461	0.466	0.19	-0.508	-0.016	-0.442	1.000

Supplementary Table 3: Intercorrelation matrix of significant molecular descriptors from PaDEL software

	SHBint4	SpMin2_Bhe	MATS8e	SssCH2
SHBint4	1.000			
SpMin2_Bhe	-0.485	1.000		
MATS8e	-0.018	0.167	1.000	
SssCH2	0.303	-0.857	-0.01	1.000

EXCLI Journal 2020;19:209-226 – ISSN 1611-2156
Received: October 18, 2019, accepted: February 19, 2020, published: February 26, 2020

Supplementary Table 4: Rational designed chemical structures of 69 new coumarin derivatives

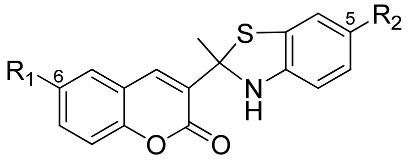

2a-2h

Compound	R_1	R_2	Predicted antioxidant activity (pIC_{50})		
			Model 1	Model 2	Model 3
**2a**	-H	-F	3.667	3.776	3.498
**2b**	-H	$-CF_3$	3.861	3.662	4.503
**2c**	-H	$-NO_2$	3.634	3.724	4.189
**2d**	$-NO_2$	-F	3.828	3.745	3.564
**2e**	$-NO_2$	$-CF_3$	3.801	3.625	4.502
**2f**	$-NO_2$	-Cl	3.828	3.289	3.401
**2g**	-CN	-Cl	3.743	3.459	3.371
**2h**	$-CF_3$	-Cl	3.716	3.167	3.378

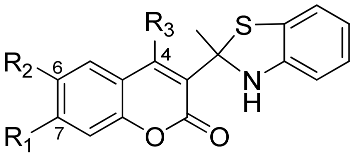

3a-3p

Compound	R_1	R_2	R3	Predicted antioxidant activity (pIC_{50})		
				Model 1	Model 2	Model 3
**3a**	-H	-Cl	-H	3.740	3.148	4.025
**3b**	-H	-F	-H	3.788	4.089	4.141
**3c**	-H	$-CF_3$	-H	3.756	3.849	3.477
**3d**	-H	$-NO_2$	-H	3.829	3.968	3.583
**3e**	-H	-CN	-H	3.546	4.140	3.741
**3f**	-OH	-Br	-H	4.075	4.575	4.564
**3g**	$-OCH_3$	-Br	-H	4.379	3.595	3.879
**3h**	$-NH_2$	-Br	-H	3.508	4.600	4.593
**3i**	$-N(CH_3)_2$	-Br	-H	3.878	3.277	3.991
**3j**	-SH	-Br	-H	3.811	4.537	4.588
**3k**	$-S(C_6H_5)$	-Br	-H	3.735	4.129	3.905
**3l**	-H	-Br	-OH	4.098	4.132	4.852
**3m**	-H	-Br	$-OCH_3$	3.791	3.017	4.352
**3n**	-H	-Br	$-NH_2$	3.660	4.182	6.340
**3o**	-H	-Br	$-N(CH_3)_2$	3.675	2.804	3.920
**3p**	$-OCH_3$	-Br	$-OCH_3$	4.451	2.948	4.491

EXCLI Journal 2020;19:209-226 – ISSN 1611-2156
Received: October 18, 2019, accepted: February 19, 2020, published: February 26, 2020

Supplementary Table 4 (cont.): Rational designed chemical structures of 69 new coumarin derivatives

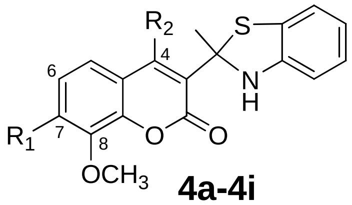

4a-4i

Compound	R_1	R_2	Predicted antioxidant activity activity (pIC_{50})		
			Model 1	Model 2	Model 3
**4a**	-OH	-H	4.286	4.362	4.729
**4b**	$-OCH_3$	-H	4.040	3.717	3.978
**4c**	$-NH_2$	-H	4.033	4.371	4.772
**4d**	$-N(CH_3)_2$	-H	3.551	3.577	4.051
**4e**	-H	-OH	4.306	3.830	4.924
**4f**	-H	$-OCH_3$	3.998	2.865	4.423
**4g**	-H	$-NH_2$	4.139	3.746	6.445
**4h**	-H	$-N(CH_3)_2$	3.456	2.601	3.969
**4i**	$-OCH_3$	$-OCH_3$	4.095	2.931	4.533

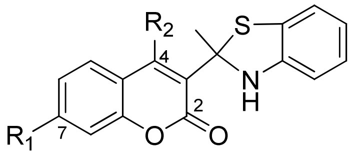

5a-5l

Compound	R_1	R_2	Predicted antioxidant activity (pIC_{50})		
			Model 1	Model 2	Model 3
**5a**	$-OCH_3$	-H	4.219	3.567	3.796
**5b**	$-NH_2$	-H	3.802	4.579	4.518
**5c**	-SH	-H	3.875	4.534	4.463
**5d**	-OH	$-CH_3$	3.804	3.705	4.410
**5e**	-OH	$- C_6H_5$	4.003	3.930	4.452
**5f**	-OH	$-OCH_3$	4.130	3.626	5.123
**5g**	-OH	-OH	4.223	4.495	5.466
**5h**	-OH	$-NH_2$	3.924	4.503	7.016
**5i**	-OH	$-N(CH_3)_2$	3.923	3.327	4.745
**5j**	$-OCH_3$	$-OCH_3$	3.892	3.517	3.055
**5k**	$-N(CH_3)_2$	-H	4.060	3.214	3.925
**5l**	$-S(C_6H_5)$	-H	3.739	4.130	3.813

EXCLI Journal 2020;19:209-226 – ISSN 1611-2156
Received: October 18, 2019, accepted: February 19, 2020, published: February 26, 2020

Supplementary Table 4 (cont.): Rational designed chemical structures of 69 new coumarin derivatives

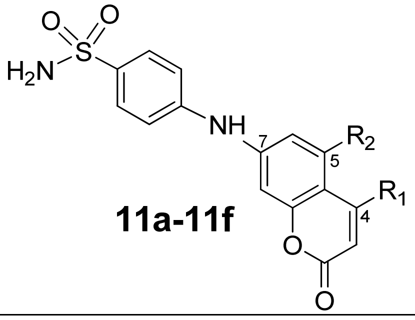

11a-11f

Compound	R_1	R_2	Predicted antioxidant activity (pIC_{50})		
			Model 1	Model 2	Model 3
**11a**	$-CH_3$	-H	3.582	4.526	3.708
**11b**	$-OCH_3$	-H	4.085	3.705	3.758
**11c**	$-C_6H_5$	-H	3.402	4.110	3.966
**11d**	$-OC_6H_5$	-H	4.198	4.317	3.775
**11e**	$-CH_3$	$-CH_3$	3.644	3.829	3.702
**11f**	$-OCH_3$	$-OCH_3$	3.970	2.610	4.364

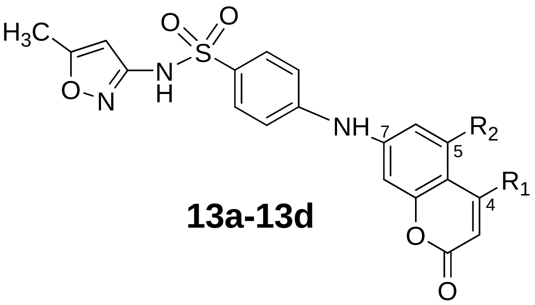

13a-13d

Compound	R_1	R_2	Predicted antioxidant activity (pIC_{50})		
			Model 1	Model 2	Model 3
**13a**	$-CH_3$	$-CH_3$	3.401	3.528	3.742
**13b**	-OH	$-CH_3$	4.035	3.903	3.607
**13c**	$-OCH_3$	$-CH_3$	4.076	3.341	3.762
**13d**	$-OCH_3$	$-OCH_3$	4.007	2.642	4.356

EXCLI Journal 2020;19:209-226 – ISSN 1611-2156
Received: October 18, 2019, accepted: February 19, 2020, published: February 26, 2020

Supplementary Table 4 (cont.): Rational designed chemical structures of 69 new coumarin derivatives

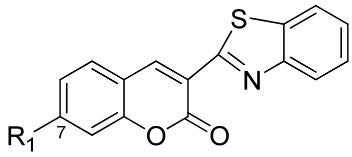

18a-18f

Compound	R_1	Predicted antioxidant activity (pIC_{50})		
		Model 1	Model 2	Model 3
**18a**	$-OCH_3$	2.958	2.820	3.037
**18b**	-OH	3.468	3.983	3.773
**18c**	$-NH_2$	2.941	4.197	3.798
**18d**	-SH	2.573	3.928	3.779
**18e**	$-S(C_6H_5)$	2.478	3.758	3.115
**18f**	$-N(CH_3)_2$	2.621	2.581	3.229

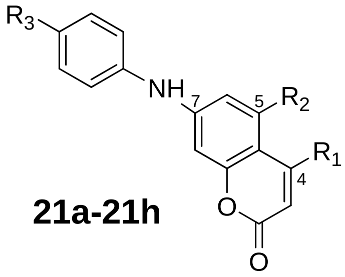

21a-21h

Compound	R_1	R_2	R_3	Predicted antioxidant activity (pIC_{50})		
				Model 1	Model 2	Model 3
**21a**	$-C_6H_5$	-H	$-OCH_3$	3.967	3.831	3.549
**21b**	$-C_6H_5$	$-OCH_3$	$-OCH_3$	4.389	2.952	4.174
**21c**	$-C_6H_5$	$-N(CH_3)_2$	$-OCH_3$	3.734	2.746	3.737
**21d**	$-C_6H_5$	-OH	$-OCH_3$	4.258	3.885	5.634
**21e**	$-C_6H_5$	$-NH_2$	$-OCH_3$	3.799	3.903	5.258
**21f**	$-C_6H_5$	-H	$-NH_2$	3.742	4.098	3.602
**21g**	$-C_6H_5$	-H	$-N(CH_3)_2$	3.918	3.745	3.584
**21h**	$-C_6H_5$	-H	-OH	3.596	4.150	3.579

EXCLI Journal 2020;19:209-226 – ISSN 1611-2156
Received: October 18, 2019, accepted: February 19, 2020, published: February 26, 2020

Supplementary Table 5: Values of significant molecular descriptors of 69 modified compounds in model 1

Compound	nArNHR	ISH	B04[O-O]	G2p
**2a**	1	0.890	0	0.1640
**2b**	1	0.879	0	0.1820
**2c**	1	0.909	0	0.1700
**2d**	1	0.849	0	0.1620
**2e**	1	0.876	0	0.1730
**2f**	1	0.849	0	0.1620
**2g**	1	0.927	0	0.1930
**2h**	1	0.873	0	0.1610
**3a**	1	0.890	0	0.1730
**3b**	1	0.862	0	0.1640
**3c**	1	0.890	0	0.1750
**3d**	1	0.877	0	0.1770
**3e**	1	0.916	0	0.1630
**3f**	1	0.885	1	0.1630
**3g**	1	0.822	1	0.1670
**3h**	1	0.923	0	0.1620
**3i**	1	0.860	0	0.1740
**3j**	1	0.885	0	0.1790
**3k**	1	0.876	0	0.1650
**3l**	1	0.923	1	0.1860
**3m**	1	0.945	1	0.1600
**3n**	1	0.916	0	0.1770
**3o**	1	0.873	0	0.1560
**3p**	1	0.830	1	0.1800
**4a**	1	0.855	1	0.1730
**4b**	1	0.925	1	0.1800
**4c**	1	0.841	0	0.1830
**4d**	1	0.896	0	0.1530
**4e**	1	0.873	1	0.1850
**4f**	1	0.914	1	0.1690
**4g**	1	0.841	0	0.1960
**4h**	1	0.918	0	0.1530
**4i**	1	0.901	1	0.1740
**5a**	1	0.846	1	0.1600
**5b**	1	0.870	0	0.1700
**5c**	1	0.870	0	0.1790
**5d**	1	0.942	1	0.1600
**5e**	1	0.896	1	0.1600
**5f**	1	0.891	1	0.1730
**5g**	1	0.877	1	0.1770
**5h**	1	0.916	1	0.1610
**5i**	1	0.907	1	0.1560
**5j**	1	0.914	1	0.1560
**5k**	1	0.797	0	0.1630
**5l**	1	0.896	0	0.1760

EXCLI Journal 2020;19:209-226 – ISSN 1611-2156
Received: October 18, 2019, accepted: February 19, 2020, published: February 26, 2020

Supplementary Table 5 (cont.): Values of significant molecular descriptors of 69 modified compounds in model 1

Compound	nArNHR	ISH	B04[O-O]	G2p
**11a**	1	0.904	0	0.1610
**11b**	1	0.877	1	0.1600
**11c**	1	0.955	0	0.1660
**11d**	1	0.881	1	0.1760
**11e**	1	0.884	0	0.1580
**11f**	1	0.896	1	0.1560
**13a**	1	0.927	0	0.1510
**13b**	1	0.913	1	0.1730
**13c**	1	0.896	1	0.1690
**13d**	1	0.895	1	0.1600
**18a**	0	0.911	1	0.1900
**18b**	0	0.840	1	0.2150
**18c**	0	0.831	0	0.1940
**18d**	0	0.905	0	0.1880
**18e**	0	0.895	0	0.1710
**18f**	0	0.858	0	0.1690
**21a**	1	0.847	0	0.1780
**21b**	1	0.831	1	0.1730
**21c**	1	0.867	0	0.1600
**21d**	1	0.869	1	0.1770
**21e**	1	0.901	0	0.1860
**21f**	1	0.884	0	0.1700
**21g**	1	0.811	0	0.1530
**21h**	1	0.931	0	0.1770

EXCLI Journal 2020;19:209-226 – ISSN 1611-2156
Received: October 18, 2019, accepted: February 19, 2020, published: February 26, 2020

Supplementary Table 6: Values of significant molecular descriptors of 69 modified compounds in model 2

Compound	D491	D467	D278	D384	D580	D461
**2a**	0.611	0.694	0.489	0	5.412	0.497
**2b**	0.630	0.636	0.505	0	5.424	0.628
**2c**	0.614	0.663	0.496	0	5.423	0.548
**2d**	0.661	0.638	0.520	0	5.448	0.427
**2e**	0.686	0.593	0.532	0	5.451	0.556
**2f**	0.640	0.649	0.520	4	5.447	0.425
**2g**	0.616	0.664	0.525	4	5.424	0.422
**2h**	0.701	0.601	0.528	4	5.446	0.325
**3a**	0.521	0.743	0.489	7	5.418	0.493
**3b**	0.507	0.754	0.489	0	5.425	0.548
**3c**	0.624	0.666	0.505	0	5.445	0.301
**3d**	0.563	0.713	0.496	0	5.445	0.395
**3e**	0.537	0.730	0.500	0	5.421	0.393
**3f**	0.499	0.757	0.531	0	5.433	0.207
**3g**	0.648	0.582	0.486	0	5.439	0.193
**3h**	0.476	0.758	0.523	0	5.428	0.189
**3i**	0.702	0.512	0.474	0	5.435	0.181
**3j**	0.501	0.734	0.531	0	5.427	0.229
**3k**	0.387	0.757	0.433	0	5.431	0.302
**3l**	0.544	0.693	0.531	0	5.459	0.547
**3m**	0.869	0.751	0.486	0	5.469	0.525
**3n**	0.535	0.736	0.523	0	5.448	0.551
**3o**	0.945	0.795	0.474	0	5.463	0.526
**3p**	0.829	0.645	0.475	0	5.487	0.345
**4a**	0.505	0.799	0.501	0	5.462	0.178
**4b**	0.602	0.773	0.454	0	5.469	0.173
**4c**	0.490	0.795	0.495	0	5.456	0.168
**4d**	0.635	0.769	0.446	0	5.465	0.167
**4e**	0.582	0.684	0.501	0	5.479	0.418
**4f**	0.796	0.739	0.454	0	5.488	0.646
**4g**	0.608	0.696	0.495	0	5.469	0.435
**4h**	0.872	0.772	0.446	0	5.482	0.770
**4i**	0.759	0.736	0.443	0	5.510	0.464
**5a**	0.632	0.627	0.462	0	5.424	0.183
**5b**	0.454	0.803	0.498	0	5.416	0.172
**5c**	0.475	0.782	0.506	0	5.415	0.205
**5d**	0.644	0.674	0.495	0	5.446	0.357
**5e**	0.454	0.804	0.440	0	5.472	0.409
**5f**	0.749	0.868	0.501	0	5.472	0.493
**5g**	0.471	0.746	0.526	0	5.461	0.297
**5h**	0.497	0.742	0.538	0	5.451	0.340
**5i**	0.837	0.909	0.489	0	5.465	0.595
**5j**	0.687	0.793	0.441	0	5.348	0.470
**5k**	0.696	0.549	0.451	0	5.421	0.176
**5l**	0.365	0.806	0.410	0	5.418	0.286

EXCLI Journal 2020;19:209-226 – ISSN 1611-2156
Received: October 18, 2019, accepted: February 19, 2020, published: February 26, 2020

Supplementary Table 6 (cont.): Values of significant molecular descriptors of 69 modified compounds in model 1

Compound	D491	D467	D278	D384	D580	D461
**11a**	0.324	0.690	0.489	0	5.539	0.022
**11b**	0.669	0.796	0.495	0	5.539	0.207
**11c**	0.325	0.823	0.425	0	5.539	0.313
**11d**	0.260	0.811	0.432	0	5.539	0.310
**11e**	0.466	0.590	0.481	0	5.539	0.244
**11f**	0.988	0.722	0.490	0	5.539	0.396
**13a**	0.414	0.533	0.452	0	5.586	0.349
**13b**	0.405	0.603	0.492	0	5.586	0.419
**13c**	0.577	0.716	0.456	0	5.586	0.439
**13d**	0.799	0.640	0.458	0	5.586	0.455
**18a**	0.669	0.385	0.433	0	5.442	0.284
**18b**	0.401	0.560	0.465	0	5.439	0.367
**18c**	0.377	0.595	0.473	0	5.436	0.299
**18d**	0.413	0.558	0.465	0	5.435	0.422
**18e**	0.288	0.704	0.371	0	5.438	0.498
**18f**	0.747	0.355	0.431	0	5.440	0.239
**21a**	0.344	0.709	0.380	0	5.412	0.308
**21b**	0.566	0.638	0.386	0	5.454	0.577
**21c**	0.645	0.654	0.387	0	5.448	0.650
**21d**	0.350	0.657	0.419	0	5.445	0.436
**21e**	0.350	0.689	0.416	0	5.437	0.481
**21f**	0.304	0.822	0.399	0	5.412	0.579
**21g**	0.352	0.627	0.381	0	5.412	0.216
**21h**	0.316	0.857	0.403	0	5.412	0.570

EXCLI Journal 2020;19:209-226 – ISSN 1611-2156
Received: October 18, 2019, accepted: February 19, 2020, published: February 26, 2020

Supplementary Table 7: Values of significant molecular descriptors of 69 modified compounds in model 3

Compound	SHBint4	SpMin2_Bhe	MATS8e	SssCH2
**2a**	4.801	1.814	0.404	0
**2b**	5.150	1.847	-0.295	0
**2c**	4.872	1.839	-0.104	0
**2d**	5.130	1.814	0.425	0
**2e**	5.480	1.847	-0.205	0
**2f**	4.415	1.818	0.424	0
**2g**	4.211	1.818	0.400	0
**2h**	4.572	1.818	0.490	0
**3a**	4.211	1.813	-0.303	0
**3b**	4.567	1.813	-0.325	0
**3c**	4.804	1.813	0.419	0
**3d**	4.649	1.813	0.268	0
**3e**	4.449	1.813	0.053	0
**3f**	7.165	1.813	-0.057	0
**3g**	4.280	1.813	-0.134	0
**3h**	6.953	1.813	-0.143	0
**3i**	4.123	1.813	-0.291	0
**3j**	6.731	1.813	-0.198	0
**3k**	4.023	1.814	-0.226	0
**3l**	7.304	1.813	-0.314	0
**3m**	5.772	1.813	-0.216	0
**3n**	13.064	1.813	-0.281	0
**3o**	4.266	1.813	-0.179	0
**3p**	5.862	1.813	-0.334	0
**4a**	7.529	1.813	-0.127	0
**4b**	4.498	1.813	-0.177	0
**4c**	7.432	1.813	-0.197	0
**4d**	4.341	1.813	-0.293	0
**4e**	7.661	1.813	-0.291	0
**4f**	6.041	1.813	-0.216	0
**4g**	13.660	1.813	-0.228	0
**4h**	4.491	1.813	-0.170	0
**4i**	6.133	1.813	-0.303	0
**5a**	4.393	1.813	-0.019	0
**5b**	7.213	1.813	0.004	0
**5c**	6.807	1.813	-0.050	0
**5d**	7.234	1.813	0.120	0
**5e**	6.914	1.813	-0.010	0
**5f**	9.160	1.813	-0.090	0
**5g**	10.790	1.813	-0.001	0
**5h**	16.903	1.813	0.063	0
**5i**	7.532	1.813	-0.143	0
**5j**	1.295	1.813	-0.097	0
**5k**	4.239	1.813	-0.192	0
**5l**	4.145	1.814	-0.100	0

EXCLI Journal 2020;19:209-226 – ISSN 1611-2156
Received: October 18, 2019, accepted: February 19, 2020, published: February 26, 2020

Supplementary Table 7 (cont.): Values of significant molecular descriptors of 69 modified compounds in model 1

Compound	SHBint4	SpMin2_Bhe	MATS8e	SssCH2
**11a**	2.048	1.884	-0.058	0
**11b**	2.106	1.874	-0.163	0
**11c**	2.058	1.911	-0.125	0
**11d**	2.112	1.882	-0.117	0
**11e**	1.974	1.893	-0.003	0
**11f**	4.384	1.875	-0.158	0
**13a**	1.965	1.899	-0.008	0
**13b**	2.017	1.888	-0.020	-0.261
**13c**	2.025	1.891	-0.063	0
**13d**	4.368	1.879	-0.125	0
**18a**	0.000	1.859	-0.102	0
**18b**	3.212	1.859	0.011	0
**18c**	2.894	1.859	-0.100	0
**18d**	2.542	1.859	-0.175	0
**18e**	0.000	1.859	-0.182	0
**18f**	0.000	1.859	-0.298	0
**21a**	2.060	1.843	-0.181	0
**21b**	4.287	1.843	-0.219	0
**21c**	2.679	1.843	-0.203	0
**21d**	9.823	1.843	-0.219	0
**21e**	8.619	1.843	-0.158	0
**21f**	2.033	1.849	-0.203	0
**21g**	1.939	1.859	-0.137	0
**21h**	2.100	1.840	-0.220	0
